# Sensory Profiling and Liking of Salami and Pancetta from Immunocastrated, Surgically Castrated and Entire Male Pigs

**DOI:** 10.3390/ani11102786

**Published:** 2021-09-24

**Authors:** Sylwia Żakowska-Biemans, Eliza Kostyra, Martin Škrlep, Marijke Aluwé, Marjeta Čandek-Potokar

**Affiliations:** 1 Institute of Human Nutrition Sciences, Warsaw University of Life Sciences (WULS-SGGW), ul. Nowoursynowska 159c, 02-787 Warsaw, Poland; 2 Agricultural Institute of Slovenia, Hacquetova ul. 17, 1000 Ljubljana, Slovenia; martin.skrlep@kis.si (M.Š.); meta.candek-potokar@kis.si (M.Č.-P.); 3 Animal Sciences Unit, Flanders Research Institute for Agriculture, Fisheries and Food (ILVO), 9090 Melle, Belgium; marijke.aluwe@ilvo.vlaanderen.be

**Keywords:** sensory profiling, check-all-that-apply, consumer panel, liking, salami, pancetta, dry-cured products, immunocastration, entire male

## Abstract

**Simple Summary:**

Alternatives to surgical castration are an important issue in pig production due to societal concerns regarding animal welfare. Castration of piglets is a common practice to avoid boar taint, an unpleasant taste (urine/fecal like) of meat from uncastrated male pigs. In view of abandoning surgical castration and introduction of new alternatives, such as raising entire male pigs and applying immunocastration, several new issues are emerging. These include boar taint occurrence in case of entire male pigs and a deteriorated raw material (meat, fat) quality, which can affect consumer perception. Therefore, more information is needed about the consumer acceptance of products from the different alternatives. In the present study, two types of dry-cured meat products were assessed to give some insights into the sensory characteristic and consumers’ liking of the products coming from immunocastrated, entire male and surgically castrated animals.

**Abstract:**

Consumer studies on acceptability of pork from immunocastrates (IC) and entire males (EM) are of primary importance, if these alternatives are to replace surgical castration (SC) of piglets. Data on the sensory traits and consumers acceptance of IC and EM meat products are still limited. Therefore, the purpose of the study was to (1) describe the sensory profile by quantitative descriptive analysis and (2) test the perception and consumer liking of salami (dry-fermented sausage) and pancetta (dry-cured belly) from EM, IC and SC animals. The consumer tests included the scaling method and check-all-that-apply. Profiling showed that EM products were scored lower in the overall sensory quality compared to IC or SC. EM products differed mainly from IC and SC in the intensity of the manure, sweat odor and flavor, persistent impression and texture (hardness, gumminess and easy to fragment). Salami samples did not differ in liking. In pancetta, the differences were significant for odor liking and visual quality (expected liking). Consumers did not perceive EM products as inferior in terms of liking, while sensory profiling indicated differences for boar taint presence and texture. Using meat originating from IC did not result in any differences in consumers acceptance as compared to products from SC.

## 1. Introduction

There is an ongoing debate in the European Union to ban surgical castration of piglets as practiced today. In 2010, on initiative of the European Commission and the Belgian Presidency, a declaration to abandon surgical castration of pigs from 1 January 2018 was drafted and signed by several actors in the European pig sector, European retailers and NGO representatives, provided that satisfactory solutions are found to the various challenges associated with the production of entire (uncastrated) male pigs [1]. Two main options are considered for transitioning away from surgical castration: raising entire male pigs (EM) or using immunocastration—a vaccine that blocks GnRH binding and thus suppresses testicular function and consequently prevents boar taint [2]. The proposed alternatives, however, raise various concerns regarding consumers’ acceptance of meat and meat products from EM and IC [1,3,4]. Consumers attach increasing importance to animal welfare and many European citizens are concerned about the welfare of farmed animals [5,6,7,8,9]. One of these ethical concerns is the castration of male piglets, which is a common practice in many countries worldwide [9].

Growing entire male pigs poses some risk by the occurrence of boar taint, an offensive odor and flavor present in meat due to skatole and androstenone that is perceived by sensitive consumers when cooking and eating tainted products [10,11]. There are several strategies to mask boar taint in meat products investigated, including smoking and adding spices [12,13], mixing tainted meat in different proportions with untainted material [14] or modifying cooking methods [15]. The risk of boar taint is high for certain products, such as those eaten warm compared to products that are consumed cold, and also for products with a higher fat percentage, as boar taint compounds are lipophilic and thus accumulate in the fat fraction [1]. Besides boar taint, there are also other meat quality issues related to the raw material from EM, including low carcass fatness, low intramuscular fat content, high fat unsaturation, issues related to water holding, pH and color deviations [13,16,17,18]. These issues may also imply that EM meat is less appropriate for processing or yields meat products of lower quality [13]. Although this alternative seems acceptable for fresh meat production due to higher lean meat deposition, it does not fulfil the requirements of the dry-cured ham processing industry [19]. An insufficient level of fat increases salt penetration and water losses, together with higher unsaturation, and has a negative impact on the final quality of the dry products [16]. There is a strong evidence that immunocastration is very effective in reducing boar taint [20,21] despite some vaccination “escapers” [22]. Even though immunocastration is an animal-welfare-friendly alternative, its market share is currently low [23]. Its development in Europe is still impaired by a strong reluctance from chain actors, based on assumed rejection of the practice by the consumers [1]. Immunocastration is considered as a good alternative to surgical castration in traditional food production because neither performance nor product quality are adversely influenced [24]. Several studies show that meat from immunocastrated pigs is accepted by the consumers [17] while consumers’ liking of EM meat is lower [25].

There are different methods applied to study consumers’ liking and acceptance of meat and meat products [11,25,26,27]. Among them, check-all-that-apply (CATA) is an approach to gather information about the sensory perception of a product and it is suggested as a valuable alternative to classical descriptive methods to determine differences among products [28,29]. The potential of the CATA method is well documented in the literature [30], but this approach has not yet been used in the research on meat products from immunocastrated and uncastrated pigs.

Therefore, the main goals of our research were twofold: (1) to determine the sensory profile of the meat products, namely, salami (dry-fermented sausage) and pancetta (dry-cured belly) originating from immunocastrated (IC), surgically castrated (SC) and entire male pigs (EM); and (2) to evaluate the perception and liking of the mentioned meat products by consumers.

## 2. Materials and Methods

### 2.1. Products

Salami (dry-fermented sausage) and pancetta (dry-cured bellies) were produced from pigs of three sex categories: EM, IC and SC. The animals used for products were raised in the experiment conducted within the ERA NET SusAN project “Sustainability in pork production with immunocastration” (SuSI) [21,31]. Pigs were crosses between Pietrain and German Landrace. Pigs of the IC group received two doses of vaccine against GnRH (IMPROVAC, Zoetis Deutschland GmbH, Berlin, Germany) at the age of 12 and 22 weeks, while pigs of the SC group were surgically castrated in the first week of life. The animals were slaughtered at 27 weeks of age and weighed 122.1 kg, 127.6 kg and 127.8 kg in groups EM, IC and SC, respectively. All pigs received the same diet containing 12.4 MJ of metabolizable energy and 15.9% crude protein, fed ad libitum (for more details, see Kress et al. [21], Kress and Verhaagh [32] and Škrlep et al. [31]). For the production of pancetta, fresh bellies (11 from each sex category) were processed in accordance with the rules of Slovenian dry-cured belly “Kraška panceta” (unsmoked product) protected by a geographical designation (PGI) [26]. The processing procedure included seven days dry salting (applying a mixture of salt and spices (black pepper and garlic) to the surface of the pieces), followed by air-drying/ripening for 12 weeks. Belly leanness was evaluated on a carcass cross section at the last rib and was 72.8%, 68.7% and 54.4% for EM, IC and SC, respectively (for more details, see Čandek-Potokar et al. [26]). The bellies were not smoked.

In the case of salami, meat of 4 pigs (one ham per pig) per sex category, with corresponding backfat, was collected. Both tissues were grinded separately using a 10 mm grinding plate and then mixed together using 80% meat and 20% fat tissue and addition to salt (2.4%), sodium nitrite (0.3%), dried garlic (0.2%) and pepper (0.2%). The meat batter was mixed thoroughly and stuffed into 40 mm in diameter collagen casings. Sausages from all three treatment groups were processed together in the same ripening chamber under standardized conditions. The ripening lasted for 74 days, after which 6 salami per batch and per treatment group were vacuum packed and stored at −20 °C until the analysis. The fat content of the finished salami was determined with near-infrared spectroscopy NIRS (NIR Systems 6500 Monochromator, Foss NIR System, Silver Spring, MD, USA) and amounted to 20.3%, 25.8% and 32.6% in EM, IC and SC salami, respectively.

### 2.2. Experiment Design and Evaluation Procedure

Analytical sensory and consumer tests were performed to evaluate salami and pancetta products. Six sets of salami and 11 of pancetta consisting of EM, IC and SC were used in the experimental design. Three triplets (EM, IC and SC) of salami and pancetta products were randomly selected for the sensory profiling with trained assessors to obtain detailed sensory characteristics of the samples. Each triplet of samples per product was assessed by consumers in one session with a 15 min break in between. The consumer test procedure consisted of an assessment of the degree of liking (scaling method) followed by a check-all-that-apply (CATA) questionnaire to evaluate consumers’ perception, hedonic and emotional response to the samples.

### 2.3. Sensory Profiling

#### 2.3.1. Training of the Assessors in Boar Taint Detection

**Figure 1:**
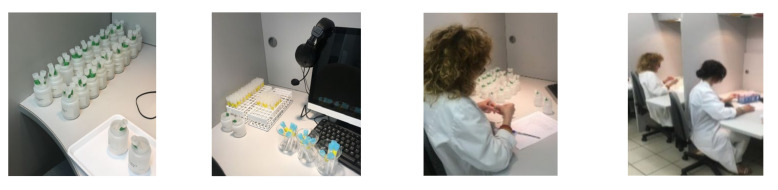
Training session related to boar taint detection by assessors participating in sensory profiling.

Prior to the sensory profiling, the assessors were checked for their sensitivity to differentiate qualitatively and quantitatively the odor of skatole (SKA) and androstenone (AND) in low (0.5 μg/g), high (5 μg/g) and very high concentrations (50 μg/g) on paper strips following the procedure elaborated by The Institute for Agricultural, Fisheries and Food Research (ILVO) in Belgium [33]. The paper strips were prepared and delivered by ILVO. Smell strips (Supplier: Carl Roth, Karlsruhe, Germany, Order no.: 1679.1) and tubes (Supplier: Carl Roth, Karlsruhe, Germany, Order no.: K938.1; Lids, Supplier: Carl Roth, Karlsruhe, Germany, Order no.: E032.1) were coded with three-digit numbers. A 20 μL drop of the appropriate solution was used to each strip and they were left to dry for 24 h in open tubes under a fume hood. After that the tubes were closed. The assessors were then trained with these spiked paper strips. The triangle method (to recognize boar taint odor), scaling method (to differentiate the intensity of the samples) and ranking method (to rank in intensity the samples and recognize the type of odor) were applied in the sensitivity tests. An example of a training session related to boar taint detection by assessors is presented in Figure 1. Finally, 10 out of 13 assessors were selected for the profiling of the samples.

#### 2.3.2. Evaluation of the Meat Products

The evaluation of the salami and pancetta samples was performed using a Quantitative Descriptive Analysis [34]. A set of 28 descriptors for salami and 30 descriptors for pancetta samples was developed during training sessions. The definitions for all the attributes were established. Products assessment included two appearance descriptors (fatness, meaty in pancetta samples), eleven odor attributes (meaty, fatty, acidic, sweet, fermentation, yeast, spicy, sweat, manure, sharp, overall odor intensity), four texture traits (hardness, gumminess, easy to fragment, coating palate with fat film), three taste descriptors (sour, salty, sweet), nine flavor traits (meaty, fatty, fermentation, yeast, spicy, sweat, manure, pungency, persistent) and overall sensory quality (the impression of the harmony of the examined attributes in the products, with no or only a slight intensity of negative notes). The impression of the harmony was perceived as the product “balanced” in the intensity of the attributes, e.g., not too sharp, hard, gummy, salty, sour, etc. according to cognitive pattern. The presence and level of sweat, manure odor and flavor as well as persistent impression were taken as negatives descriptors. The intensity of descriptors was assessed on an unstructured 10 cm line scale, ranging from low (value 0, the left side) to high intensity (value 10, the right side of the scale). Profiling of the examined products was performed over a period of six days (one set × three samples of products × two replications per day). Twenty individual results for each salami and pancetta sample were used for statistical analysis and the interpretation of the data.

### 2.4. Consumers Tests

#### 2.4.1. Participants

The consumer study involved 105 young adults (21–25 years old), 75% female and 25% male residents of Warsaw, Poland. Consumer assessments were conducted at set times of the day and subsequent days. A maximum of 10 consumers participated per session. Only those participants consuming pork and being responsible for shopping decisions were selected to participate in the study. 11.4% participants declared they consumed pork meat more than 4 times a week, 11.4% consumed pork 2–3 times per week, 47.6% consumed pork 1–2 times per week and 29.6% less than once per week. Most of the participants (60.9%) had a secondary education and 39.1% declared to have higher education. The majority of participants were familiar with the term “piglet castration” (90.2%) and “boar taint” (86.7%).

#### 2.4.2. Scaling Method

Consumers evaluated both products in terms of expected liking, odor liking, taste/flavor liking, texture liking and experienced (overall) liking. Expected liking was based on the external appearance of the products and associations of consumers regarding the overall perception of the sensory characteristics. The consumers’ liking of all attributes of the products was assessed using a 9-point hedonic structured scale with the following categories: dislike extremely, dislike very much, dislike moderately, dislike slightly, neither like nor dislike, like slightly, like moderately, like very much, like extremely [35].

Additionally, consumers were asked to indicate their willingness to buy (WTB) for each sample using a 9-point scale (1 = “would definitely not buy” and 9 = “would definitely buy”).

#### 2.4.3. Check-All-That-Apply (CATA) Questions

To get more insight into key sensory and emotional/hedonic attributes, consumers performed a CATA task for all salami and pancetta samples. CATA is a multiple-choice style question where participants are presented with a list of sensory attributes relevant to the product category being investigated and are asked to select all the options they can detect when tasting a product (all those they believe that apply) [36]. According to Ares and Varela [30], the terms in CATA might include, e.g., sensory attributes, hedonic responses and emotional responses. The attributes used in the CATA questions to evaluate salami and pancetta products were provided by consumers during preliminary tests as well as selected based on a literature review [29,37,38]. The CATA questions included 25 attributes of the examined products, including 7 flavor descriptors, 3 related to taste and 3 describing texture, whereas 12 terms had an emotional and hedonic meaning (Table 1). The list of attributes and the principle of product evaluation using the CATA questions were explained prior to the sample evaluations. In this way, consumers had the possibility to recall and check all attributes that could apply throughout the assessment. Participants were asked to try the products and then indicate all the attributes considered appropriate with the sample being evaluated.

### 2.5. Sample Preparation and Presentation

#### 2.5.1. Preparation of the Product Samples

The individual product samples of salami (two slices, 2.0 mm thick) and pancetta (one slice, 1.5 mm thick) were put into coded (3-digit numbers) plastic containers (200 mL) and covered with lids. A meat slicer was applied to cut the products. To standardize and check the thickness of the first slices of sample (regardless of the type of product) a Vernier caliper was used.

#### 2.5.2. Presentation of the Products Samples

The order of the sample presentation to the trained assessors and consumers was balanced to reduce possible carry-over effects between products. In profiling, first a salami assessment was done for three consecutive days, and after one day off, the evaluation of the pancetta sample set was carried out. The consumer tests were performed in accordance with the pre-established rating schedule. In each session consumers evaluated 3 salami and 3 pancetta samples from EM, IC and SC animals. The interval between the salami and pancetta samples was approximately 8–10 min.

**Figure 2:**
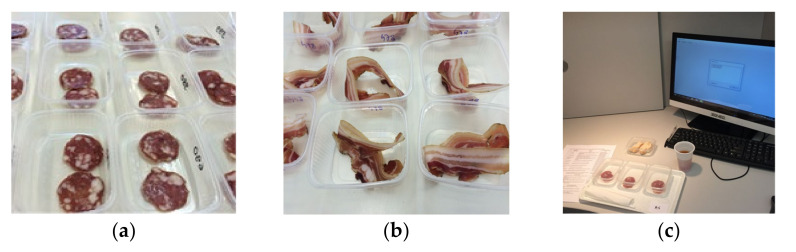
An example of the preparation and presentation of the samples for sensory profiling: (**a**) salami samples, (**b**) pancetta samples, (**c**) preliminary evaluation session with salami (sensory booth).

The trained assessors and consumers received samples in a random order at room temperature (21 ± 2 °C) and evaluated them under white bulb light. Unsweetened tea (at the temperature of approximately 50 °C) and a piece of matzah were given as a taste neutralizer between the samples. An example of the preparation and presentation of the samples for profiling is given in Figure 2a–c.

#### 2.5.3. Testing Conditions

All the sessions involving experts and consumers were performed in an accredited sensory laboratory, equipped with 10 individual testing booths, that met all the necessary requirements [39].

### 2.6. Statistical Analysis

The data was analyzed using XLSTAT statistical software (2017; Addinsoft, France, Paris) and SAS 9.4 software (SAS Institute Inc., Cary, NC, USA).

Profiling results were analyzed by two-way ANOVA with interactions to find the differences in intensity of the attributes, respectively, for the salami and pancetta samples considering the products, assessors and their interactions as fixed variables. Means were compared using Fisher’s LSD significant test. A threshold probability level considered for statistical significance was at *p* < 0.05. A Principal Components Analysis (PCA) was performed to determine the similarities and differences in the sensory characteristics of the examined samples.

Chi-square and Cochran’s Q tests were used, respectively, to determine whether the proportions chosen by consumers for all attributes and for individual terms of the CATA question varied depending on the sex category (EM, IC and SC) for each type of product. If significant differences were identified among the variables, post hoc multiple pairwise comparisons were performed using McNemar’s test with Bonferroni alpha adjustment. Correspondence analysis, based on chi-square distance, was used to visualize associations between the CATA attributes and the evaluated products.

The data on consumers’ liking were analyzed using one-way ANOVA with a post-hoc Fisher’s LSD significant test. To determine the differences between the expected and experienced liking, repeated measures analysis was applied with group and repetition as the fixed effect, and their interaction.

## 3. Results

### 3.1. Sensory Properties of Salami and Pancetta Products (Quantitative Descriptive Analysis Results)

The mean values for the sensory attributes of EM, IC and SC salami and pancetta products are shown in Table 2. It was found that the evaluated samples varied significantly in the intensity of several attributes, as well as in their overall sensory quality.

The salami samples (EM, IC and SC) differed in terms of odor attributes, such as yeast, sweat, manure and sharpness. The differences were also observed in evaluation of texture descriptors such as hardness, gumminess and easiness of fragmentation. The flavor of the salami was differentiated by meaty, sweat, manure attributes and persistent impressions. Significant differences were also noted for overall sensory quality.

The highest mean values were observed for sweat, manure odor and flavor as well as sharp odor and persistent impression in EM salami samples. The texture of the EM salami samples was also scored the highest in hardness and gumminess, whereas the mean value for easiness of fragmentation was the lowest and significantly different from IC and SC. The EM samples were similar to IC in attributes such as acidic and yeast odor. The EM variant obtained the lowest score in terms of overall sensory quality.

Pancetta samples differed in sweat and sharp odor and texture attributes such as hardness, gumminess and easiness of fragmentation. The flavor of pancetta differed among the sex categories in attributes such as fatty, salty, spicy, sweat, manure and persistent impressions. The observed differences in the intensity of the mentioned descriptors influenced the overall sensory quality of pancetta.

The sweat and sharp odors were the most perceptible in the EM sample. The pancetta samples originating from EM also scored the highest in texture attributes such as hardness and gumminess. The differences in terms of hardness were significant as compared to IC and SC, whereas EM samples were similar in gumminess and easiness of fragmentation to IC but differed from SC. The sweat, manure flavor and persistent impressions were the highest in the EM sample and differed significantly from IC and SC. The sample coming from EM animals was perceived as the lowest in fattiness and spiciness, whereas the IC and SC samples were similar in the intensity of these attributes. The saltiness of the IC sample was the highest and significantly different from EM and SC. The overall sensory quality was the lowest for EM samples, whereas IC and SC were considered as similar.

Pancetta was also assessed by trained assessors in terms of appearance attributes such as fattiness and meaty. The results indicated that samples from EM animals were perceived as being less fatty (sensory scores: EM 5.4 c.u., IC 6.4 c.u., SC 6.3 c.u.) and more meaty (EM 3.9 c.u.) compared to IC (3.3 c.u.) and SC (2.9 c.u.) samples.

**Figure 3:**
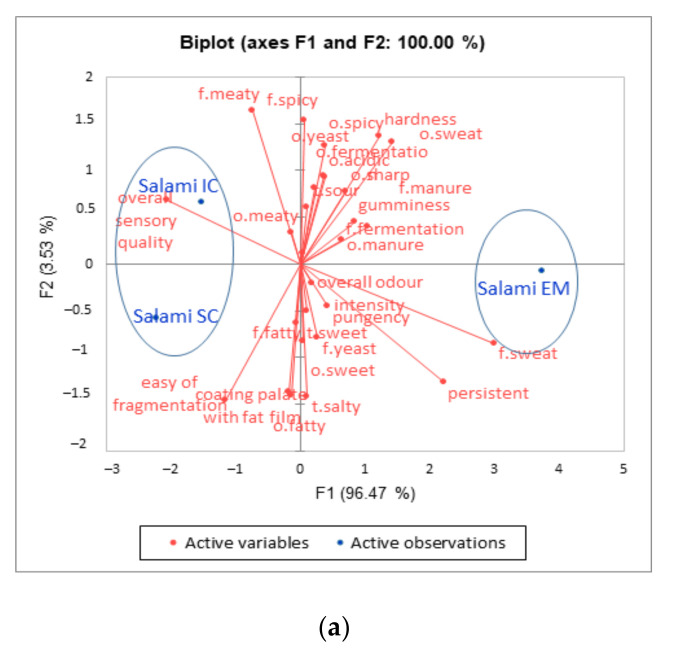

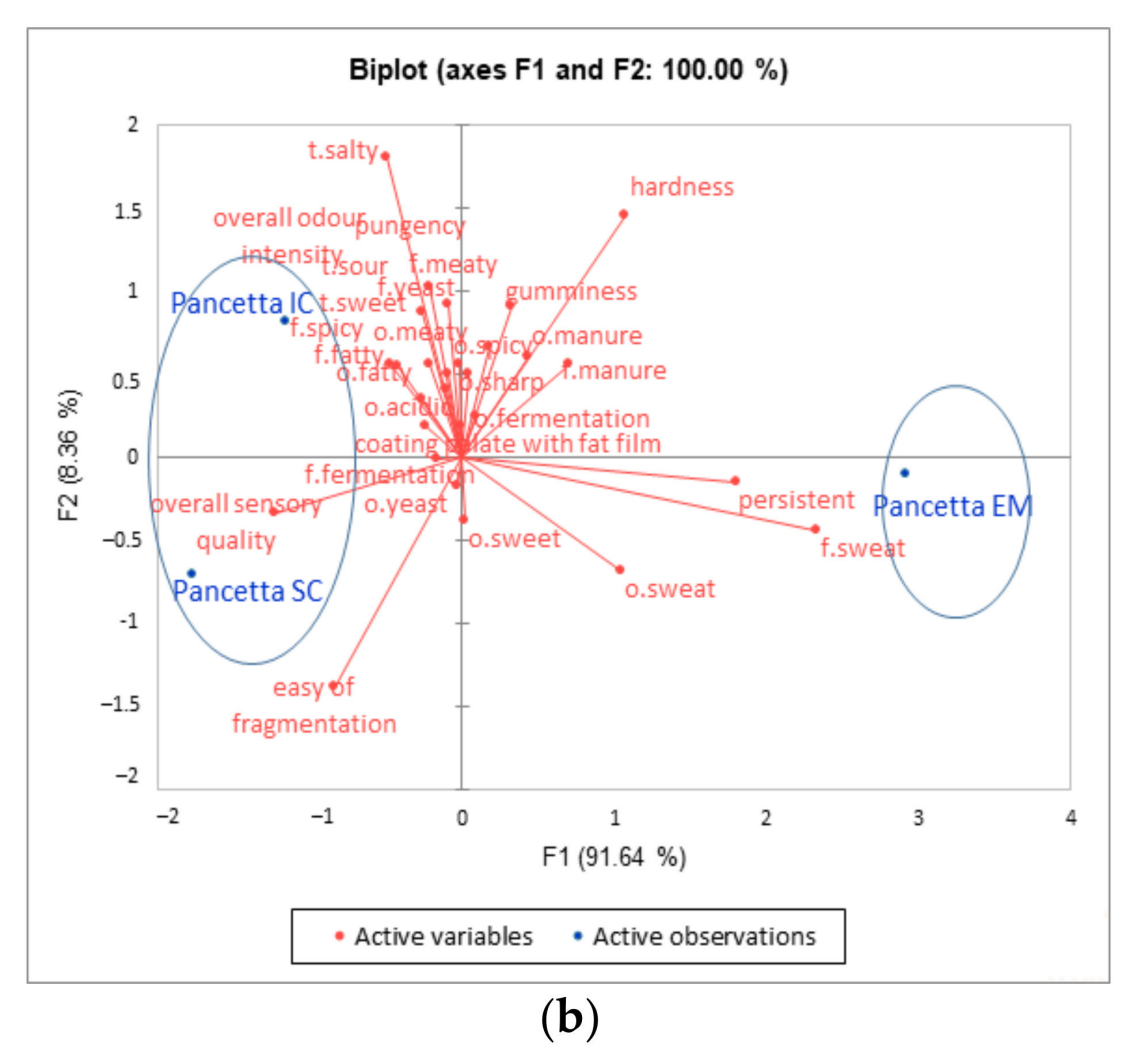
Principal component analysis (PCA) of the EM, IC and SC salami (**a**) and pancetta (**b**) sensory attributes. EM = entire males; IC = immunocastrates; SC = surgical castrates.

The results of profiling of the examined products are displayed in Figure 3a,b as a PCA biplots. Relatively close similarity in sensory characteristics of IC and SC and dissimilarity in relation to EM is clearly marked. Almost all of the variability of the salami (96.47%) and pancetta (91.64%) samples was attributed to the First Principal Component (F1, horizontal axis).

In the case of salami samples, F1 was characterized by acidic, sharp, spicy odor, fermentation odor and flavor, yeast odor and flavor, sweat odor and flavor, manure odor and flavor as well as an overall odor intensity, hardness, gumminess, sour taste, sweet taste, pungency and persistent impressions with a positive loading, while a meaty odor and flavor, ease of fragmentation and overall sensory quality displayed a negative loading. The IC and SC salami were far from the EM sample, which was positioned close to the attributes negatively associated with overall sensory quality, such as sweat flavor, persistent impression, manure odor and flavor, gumminess and hardness.

It was found that F1 for the pancetta samples was represented by fermentation odor, sweat odor and flavor, sharp odor, hardness, gumminess, manure flavor and a persistent sensation with positive loading, while attributes such as fatty odor, easy to fragment, coating palate with fat film, fatty flavor, sour taste, sweet taste, fermentation flavor, spicy flavor and overall sensory quality displayed a negative loading. As in the case of salami, pancetta originating from SC and IC animals were at a distance from the EM sample positioned near sweat odor and flavor and persistent feature.

### 3.2. Sensory and Hedonic Perception of Salami and Pancetta Products by Consumers (CATA Results)

The frequency of CATA terms used by consumers to describe the sensory and hedonic attributes of EM, IC and SC salami and pancetta samples are summarized in Table 3. There were statistically significant differences between the EM, IC and SC salami in the frequency of using the terms describing a taste/flavor such as meaty and irritating. IC salami was more frequently described as meaty and differed significantly from EM, whereas the differences between IC and SC were not significant. EM salami was more often described as irritating comparing the IC and SC that were perceived similar. In terms of texture, the EM samples were more often considered as gummier and differed significantly from IC and SC. The perception of hardness also differentiated the sex categories. The EM samples were perceived as similar in hardness to IC but different from SC, whereas the differences between IC and SC were not significant.

Pancetta samples differed between the EM, IC and SC variants in the frequency of using the terms fatty, hardness and interested. Consumers perceived EM samples as less fatty compared to the IC and SC variants. The samples originating from SC were considered the most frequently as fatty. The texture of EM pancetta differed in hardness from SC but the differences between EM and IC were not significant. The EM samples also more often evoked an emotional association with the term interested and differed significantly from IC and SC.

**Figure 4:**
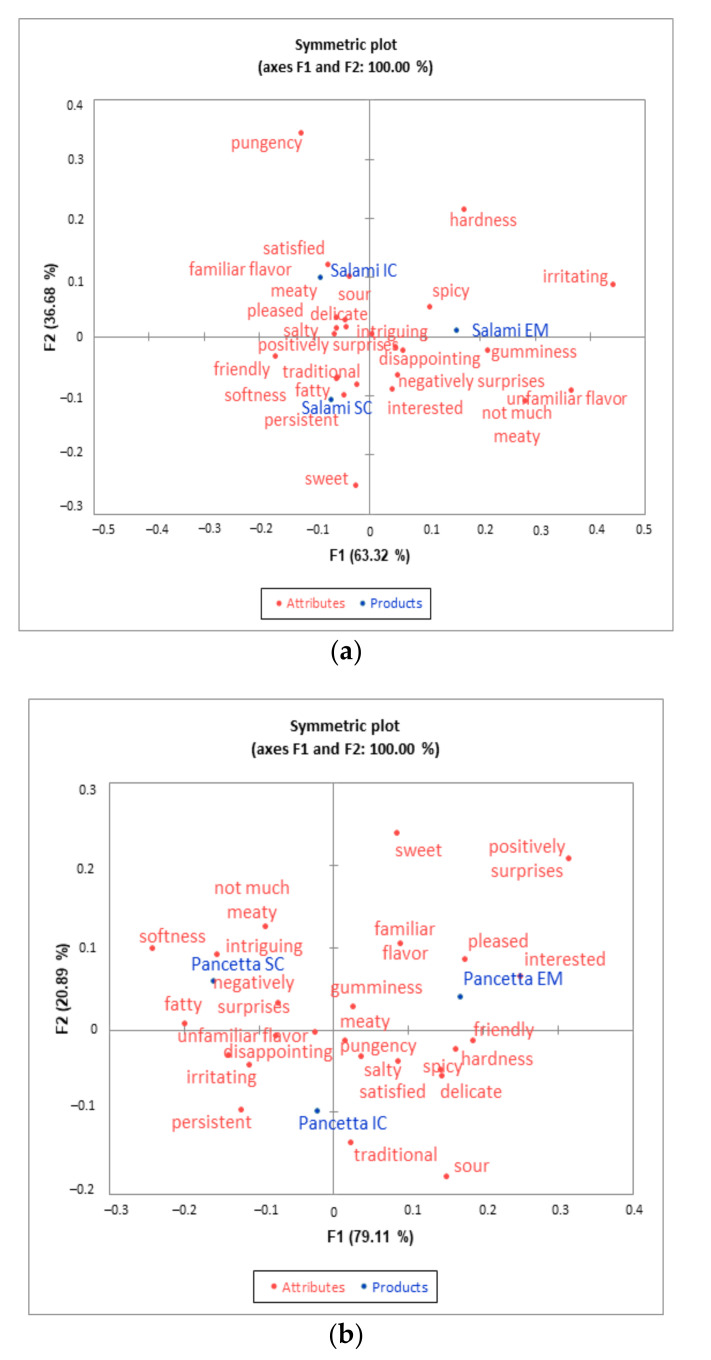
Representation of the salami (**a**) and pancetta (**b**) samples and the attributes in the first and second dimensions of the correspondence analysis obtained from the CATA total frequency counts.

The correspondence analysis of the CATA results included in Figure 4a,b illustrates how the examined attributes in both products are located. The attributes that best described the salami from EM were gumminess, spicy as well as irritating, not much meaty and an unfamiliar flavor, whereas IC salami was more related to meaty, sour, familiar flavor, satisfied and pleased. The SC salami sample was more linked to fatty, softness, traditional and friendly terms.

Pancetta originating from EM animals was more associated with attributes describing emotional features (interested, pleased and familiar flavor). The IC variant was more related to persistent and traditional attributes, whereas pancetta from SC animals was more linked to the terms fatty, softness, negatively surprises and intriguing.

### 3.3. Liking of Salami and Pancetta Samples

The mean liking and willingness to buy salami and pancetta are presented in Table 4. There were no significant differences in consumers’ liking between the EM, IC and SC samples of salami.

In terms of pancetta, the differences were significant only in odor liking. EM pancetta samples were scored significantly higher as compared to the IC and SC samples. Generally, the salami samples were more appreciated by participants than pancetta and generated a higher willingness to buy. The EM, IC and SC salami did not differ significantly in expected and experienced overall liking. In contrast the differences were noted for EM, IC and SC pancetta but only in the expected overall liking.

**Figure 5:**
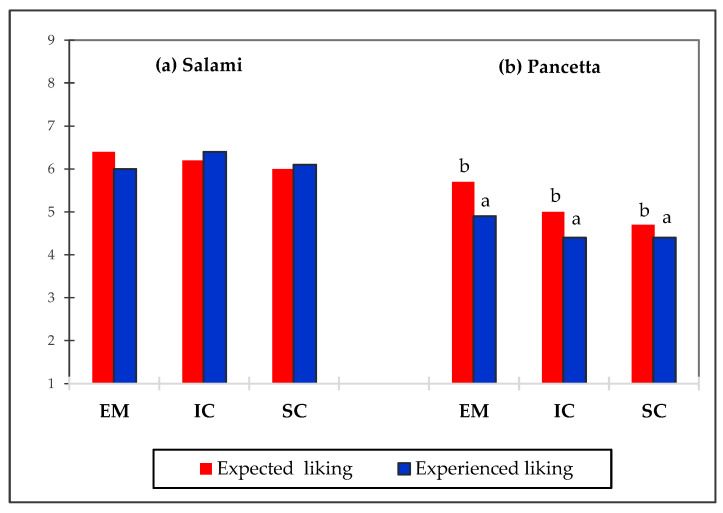
Comparison of the expected and experienced liking of the EM, IC and SC salami (**a**) and pancetta (**b**).

Liking of products differed when comparing the mean scores for pancetta samples in both evaluation condition. The differences between expected and experienced liking for are illustrated in Figure 5. All pancetta samples were scored higher in the expected liking, whereas differences for salami were insignificant.

## 4. Discussion

Many studies have shown that the sensory properties of meat products affect the consumer’s liking [40,41] and determine their success or failure in the food market [42]. Abandonment of surgical castration raises many concerns among stakeholders with regard to product quality and, particularly, consumers’ acceptance [1,9]. The present study identifies the sensory profiles of meat products coming from EM, IC and SC animals by applying QDA, contributing to a better understanding of consumers’ perception and liking of such products, also by combining CATA with the scaling method.

According to Murray et al. [43], descriptive analysis (DA) is one of the most flexible, powerful, sophisticated and widely used tools in sensory science to determine the profile of products. Sensory profiling of salami and pancetta performed by the assessors sensitive to boar taint revealed significant differences between the products coming from EM, IC and SC animals. The EM meat products represented a higher intensity of sweat, manure odor and flavor, sharp odor and persistent impression. Simultaneously, EM products showed the hardest texture and the lowest overall sensory quality. These findings are in line with the study of Čandek-Potokar et al. [44] on the quality of Slovenian dry-cured ham from EM, IC and SC animals. Harder texture of EM pancetta could be related to lower fatness, as shown in the study of Čandek-Potokar et al. [26] where the same raw material was used. Again, the main reason being the lower fat content, resulting in higher processing losses and, finally, a drier, harder and chewier product [45,46].

In our study, IC and SC products revealed quite similar sensory properties, being at a distance from EM, which is in line with other research on dry-cured hams [44,47], dry-fermented sausages [48] and pork bellies [49]. Škrlep et al. [31] concluded that when considering the use of immunocastration as an alternative, the benefits to surgical castration can be expected for carcass quality, while, compared to EM, the main advantage is a better quality of meat due to lower meat toughness.

Previous studies on the consumers’ sensory assessment of EM meat shown that there is a high rejection risk, due to the presence of boar taint [50,51,52]. On the other hand, it should be noted that boar taint perception differs among various examined meat products [52,53]. Fresh meat products, such as loins and cutlets, are subject to a higher rejection risk, especially when heated, compared to processed meat products such as dry fermented sausage and cooked and dry-cured ham [50,54,55,56].

Using the CATA method enabled to go beyond the attributes related to the presence of boar taint and to identify other descriptors discriminating between the sex variants. There were significant differences in the perception of the sensory attributes, such as meaty, fatty, irritating, hardness and gumminess, whereas emotional attributes did not differentiate the perception of the samples except for interested. The results of CATA showed that fat content and texture contribute to consumers’ acceptance of dry cured products. Our study confirmed that CATA questions represent a good alternative to study the sensory characteristics of meat products, as revealed by Jorge et al. [29] and Kessler et al. [37].

Overall, salami samples were perceived more positively by consumers than pancetta, which was also reflected in the results of the degree of liking and willingness to buy. There were no clearly marked differences in liking of, taste/flavor and texture as well as willingness to buy between the EM, IC and SC samples (except expected overall liking). Interestingly, EM, IC and SC salami received very similar liking scores for most of the attributes, whereas significant differences were noted for pancetta. EM pancetta was scored higher compared to IC and SC for most of the attributes, which indicates the influence of other sensory factors/cues than boar taint on the results [25]. The data from the expert profiling indicated that the pancetta samples from EM animals had less visual fat and more visual meat compared to IC and SC samples, also a lower carcass fatness and higher belly leanness % (as observed on the same animals in the study of Škrlep et al. [31]). As a result, pancetta from EM was perceived as leaner, which could affect consumers’ liking scores. This corroborates with the results of the Slovenian consumer panel performed with the same pancetta samples by Čandek-Potokar et al. [26]. A potential explanation may be related to the fact that consumers prefer leaner pieces [57] even when boar taint is present in the product. On the other hand, it should be taken into account that only a certain part of consumers may be sensitive to boar taint [58]. In the raw material that was used for salami and pancetta, boars had on average 2.53 AND µg/g fat, whereas the IC had AND levels below the limit of detection (<0.24 µg/g fat). The level of SKA was relatively low (0.037 µg/g fat) and detected only in EM (for more details, see Kress et al. [21]). EM salami (finished product) had comparable boar taint levels, i.e., 2.78 µg/g fat AND and 0.047 SKA (data not shown). In the pancetta samples (fresh fat tissue), the levels of AND were higher (8.45 µg/g fat on average) while the SKA level was below detection (0.045). In IC and SC both substances were below the detection levels. Mörlein et al. [14] found that there was no effect of androstenone and skatole concentrations in the raw material on the liking of processed meat products [57].

In terms of expected liking, EM pancetta was more liked as compared to the IC and SC samples, while in the experienced liking there were no significant differences between EM, IC and SC. It was also noted that in experienced liking all pancetta samples were scored significantly lower as in expected liking. These findings confirm that visual perception play a crucial role in creating sensory expectations for products [42,59]. The discrepancies between consumer’s expectations and the experience with regard to the pancetta samples could have been induced by the contrast effect [60]. According to the literature, when participants perceive a large differences between expected and experienced stimulus, this generates surprise, which leads to a contrast effect rather than assimilation (minimization differences between the perception of the product and its expectation) [61,62].

Our study has also some limitations because the participants were very homogenous regarding the age categories, and the sample is not representative for the overall population. We did not study the different age groups in the comparison of differences in sensory characteristics between EM, IC and SC; but, it can be expected that their sensitivity to androstenone is varies, as it has been shown that it is higher in females than males, and also at a younger age [63,64]. Other studies should focus on the age-related differences in sensitivity to boar taint compounds and their impact on sensory perception and liking of meat products.

## 5. Conclusions

Sensory profiling indicated that mainly EM products differed from IC and SC based on attributes related to boar taint and texture. The data on consumer liking revealed that the EM, IC and SC salami samples did not differ. In terms of pancetta the differences were significant only for odor liking with EM being significantly different from IC and SC. Consumers’ expected liking of EM pancetta compared to IC and SC was higher due to the lower fat content of the product, whereas the differences in the experienced liking were not significant. Using meat originating from IC did not result in significant differences in consumer acceptance as compared to products from SC. The research approach we applied allows to draw direct comparisons between the results of sensory profiling, liking and CATA, to deepen the understanding of the factors that influence consumers’ perception and acceptance of meat products from IC versus EM and SC. Our study also confirmed that differences in consumers’ perception of EM, IC and SC products are determined by other sensory cues than those related to the presence of compounds responsible for boar taint. Therefore, it is important to consider the product-related differences in dry-cured meats and combine different sensory research methods. Consumer sensory studies are crucial to identify the multifaceted factors contributing to the acceptance of meat products coming from production systems alternative to surgical castration and to provide stakeholders with the relevant information on the pros and cons of the proposed alternatives.

## Figures and Tables

**Table 1:** Attributes used in check-all-that-apply (CATA) questionnaire for salami and pancetta products.

Flavor	Taste	Texture	Hedonic/Emotional
meaty	salty	gumminess	familiar flavor
not much meaty	sour	softness	unfamiliar flavor
fatty	sweet	hardness	delicate
spicy			pleased
pungency			disappointing
persistent			positively surprises
irritating			intriguing
			negatively surprises
			satisfied
			interested
			friendly
			traditional

**Table 2:** Sensory profiling of the EM, IC and SC salami and pancetta products (*n* = 20).

Attributes	Salami				Pancetta			
	EM	IC	SC	*p-*Value	EM	IC	SC	*p-*Value
Odor								
odor meaty	4.6	4.8	4.7	0.705	4.0	4.1	3.9	0.465
odor fatty	4.3	4.3	4.7	0.175	5.4	5.8	5.7	0.103
odor acidic	3.3	3.0	2.8	0.068	1.8	1.8	1.8	0.910
odor sweet	1.2	1.0	1.2	0.384	1.1	0.9	1.1	0.418
odor fermentation	3.4	3.3	3.1	0.293	2.1	2.1	2.0	0.644
odor yeast	1.9 ^b^	1.6 ^ab^	1.4 ^a^	0.015	1.1	1.1	1.2	0.811
odor spicy	2.7	2.5	2.2	0.063	1.9	1.9	1.7	0.530
odor sweat	3.0 ^b^	1.7 ^a^	1.2 ^a^	<0.001	2.1 ^b^	0.8 ^a^	0.9 ^a^	<0.001
odor manure	1.5 ^b^	0.9 ^a^	0.8 ^a^	0.002	0.9	0.9	0.6	0.065
odor sharp	3.1 ^b^	2.5 ^a^	2.2 ^a^	0.006	3.0 ^b^	2.7 ^ab^	2.4 ^a^	0.015
overall odor intensity	5.6	5.4	5.4	0.740	5.3	5.5	5.3	0.413
Texture								
hardness	6.4 ^b^	5.4 ^a^	4.9 ^a^	<0.001	7.3 ^c^	6.5 ^b^	5.7 ^a^	<0.001
gumminess	6.4 ^b^	5.6 ^a^	5.4 ^a^	<0.001	7.1 ^b^	7.0 ^ab^	6.6 ^a^	0.047
easy of fragment	3.8 ^a^	4.8 ^b^	5.3 ^b^	<0.001	2.2 ^a^	2.7 ^a^	3.4 ^b^	<0.001
coating palate with fat film	5.5	5.5	5.8	0.288	6.2	6.5	6.4	0.389
Flavor and taste								
flavor meaty	4.8 ^a^	5.8 ^b^	5.5 ^b^	<0.001	4.2	4.5	4.2	0.187
flavor fatty	5.0	5.0	5.1	0.799	5.6 ^a^	6.2 ^b^	6.0 ^b^	0.019
taste sour	3.5	3.5	3.4	0.716	1.6	2.0	1.7	0.068
taste salty	5.3	5.0	5.3	0.403	4.5 ^a^	5.4 ^b^	4.8 ^a^	0.007
taste sweet	1.0	0.9	1.0	0.380	0.9	1.2	1.0	0.087
flavor fermentation	3.4	3.4	3.4	0.972	1.8	2.0	2.0	0.513
flavor yeast	1.7	1.3	1.4	0.164	0.9	1.1	1.0	0.232
flavor spicy	3.5	3.6	3.3	0.483	2.1 ^a^	2.7 ^b^	2.6 ^ab^	0.037
flavor sweat	4.7 ^b^	1.5 ^a^	1.3 ^a^	<0.001	3.4 ^b^	0.9 ^a^	0.7 ^a^	<0.001
flavor manure	1.8 ^b^	0.8 ^a^	0.6 ^a^	<0.001	1.5 ^b^	0.9 ^a^	0.6 ^a^	<0.001
pungency	4.2	3.7	3.8	0.254	2.4	2.8	2.5	0.144
persistent	4.4 ^b^	1.9 ^a^	1.9 ^a^	<0.001	3.5 ^b^	1.6 ^a^	1.4 ^a^	<0.001
overall sensory quality	3.0 ^a^	5.2 ^b^	5.3 ^b^	<0.001	3.2 ^a^	4.4 ^b^	4.7 ^b^	<0.001

EM = entire males; IC = immunocastrates; SC = surgical castrates. ^a–c^ Different superscripts in indicate significant differences between the product (separately for salami and pancetta) and the sex category for each attribute (*p* < 0.05).

**Table 3:** Frequency (%) of the use of the sensory terms included in the CATA question for the evaluation of the salami and pancetta products (*n* = 105).

Attributes	Salami				Pancetta			
	EM	IC	SC	*p-*Value	EM	IC	SC	*p-*Value
Flavor and taste								
meaty	69 ^a^	82 ^b^	76 ^ab^	0.049	53	51	48	0.703
not much meaty	17	8	12	0.157	31	26	37	0.162
fatty	48	51	60	0.170	51 ^a^	66 ^b^	79 ^c^	<0.001
salty	61	71	70	0.138	67	65	57	0.199
sour	14	16	15	0.905	12	13	7	0.260
sweet	9	7	13	0.229	6	3	5	0.558
spicy	50	42	38	0.140	31	27	20	0.132
pungency	8	14	6	0.094	8	8	8	1.000
persistent	15	15	19	0.633	30	41	39	0.127
irritating	13 ^b^	6 ^a^	5 ^a^	0.042	21	26	26	0.535
Texture								
gumminess	50 ^b^	31 ^a^	35 ^a^	0.006	55	49	48	0.437
softness	31	33	39	0.426	14	16	24	0.078
hardness	26 ^b^	23 ^ab^	13 ^a^	0.042	53 ^b^	43 ^ab^	33 ^a^	0.006
Hedonic/Emotional								
familiar flavor	35	42	33	0.172	19	13	15	0.326
unfamiliar flavor	10	4	6	0.155	2	24	25	0.832
delicate	24	27	26	0.862	25	22	16	0.148
pleased	48	56	54	0.460	28	18	18	0.092
disappointing	18	16	17	0.928	30	39	39	0.241
positively surprises	18	18	18	1.000	12	5	6	0.129
intriguing	14	12	13	0.902	13	13	18	0.396
negatively surprises	12	10	12	0.852	30	30	34	0.701
satisfied	27	36	27	0.191	12	11	9	0.727
interested	40	33	42	0.321	34 ^b^	21 ^a^	18 ^a^	0.004
friendly	24	35	37	0.083	22	17	13	0.234
traditional	33	32	39	0.437	20	24	17	0.368

^a,b^ Different superscripts indicate that the frequency for each attribute between product (separately for salami and pancetta) and the sex category differed significantly according to Cochran’s Q test.

**Table 4:** Mean liking scores and willingness to buy salami and pancetta product samples (*n* = 105).

Liking Attributes	Salami				Pancetta			
	EM	IC	SC	*p-*Value	EM	IC	SC	*p-*Value
odor liking	6.0	6.2	6.2	0.642	5.6 ^b^	5.0 ^a^	4.8 ^a^	0.010
flavor/taste liking	6.2	6.4	6.1	0.474	5.1	4.6	4.6	0.095
texture liking	6.3	6.7	6.2	0.053	4.6	4.4	4.4	0.703
expected overall liking	6.4	6.2	6.0	0.170	5.7 ^b^	5.0 ^a^	4.7 ^a^	0.001
experienced overall liking	6.0	6.4	6.1	0.220	4.9	4.4	4.4	0.087
willingness to buy	5.5	5.9	5.5	0.393	4.1	3.6	3.5	0.081

^a,b^ Different superscripts indicate significant differences between the product (separately for salami and pancetta) and the sex category (*p* < 0.05).

## Data Availability

Not applicable.
